# The Role of Beliefs, Pride, and Perceived Barriers in Decision-Making Regarding Purchasing Value-Added Pulse Products among US Consumers

**DOI:** 10.3390/foods11060824

**Published:** 2022-03-13

**Authors:** Sun-Hwa Kim, Wan-Yuan Kuo

**Affiliations:** 1 Hospitality, Tourism, and Restaurant Management, Health and Human Development, Montana State University, Bozeman, MT 59717, USA; 2 Sustainable Food System, Health and Human Development, Montana State University, Bozeman, MT 59717, USA; wanyuan.kuo@montana.edu

**Keywords:** value-added pulse product, pride, theory of planned behavior, pulses

## Abstract

This study explores the underlying psychological structure of purchasing value-added pulse products. It expands the theory of planned behavior (TPB) model by incorporating an emotional factor and explains consumers’ attitudes and subsequent behavioral intentions in the context of value-added pulse products (VAPPs). The study results showed the significant effect of pride on the purchase intention of value-added pulse products, as well as the moderating effect of perceived barriers on some of the relationships among the variables. Although value-added pulse products are emerging as a means of income maximization in the agri-food industry, there is a lack of understanding about consumers who purchase these products. This study fills the gap by developing a research framework for agriculture-related businesses. The findings may provide further insights into consumers’ attitudes and behaviors in consuming agri-foods, thereby assisting pulse producers and marketers to develop a more effective marketing strategy.

## 1. Introduction

Pulses (e.g., beans and chickpeas) have significant health and nutritional benefits such as lowering calories or cholesterol and providing dietary fiber [1,2]. However, the consumption of pulses has been limited in the United States [1]. For instance, their daily consumption in the United States is 9 g, while people in Asian countries consume 110 g [3]. Low consumption of pulses is largely attributed to the time and effort required to prepare and cook them, since they are not generally consumed raw. These challenges (e.g., length of time to cook) call for an opportunity to develop value-added pulse products [4] because value-added pulse products (VAPPs) may alleviate such challenges in the U.S., thereby increasing pulse consumption [1].

Research regarding VAPPs has not fully emerged in the field of agri-food business [1,5]. Existing research on pulses (pulse research) has focused on the material properties and functionalities of diet [6,7] and Asian countries where pulses are commonly used in traditional dishes, largely missing the opportunities in the U.S. to increase pulse consumption through understanding consumers who may purchase VAPPs [1]. This is problematic for the agri-food business, as consumer interest in VAPPs has not only increased significantly but is further expected to grow [4,8,9] in the U.S. [10]. Although the agri-food industry has called for research efforts to understand how consumers might behave in purchasing VAPPs, attempts to respond to such research inquiries are still largely unavailable for pulses producers and the agri-food industry [10] to create new markets, promote VAPPs strategically, and sustain consumers’ interest in VAPPs [11]. As such, a theoretical and empirical investigation of the underlying structure affecting consumers’ consumption in VAPPs is required [12]. There are some discussions about the demographic characteristics of consumers who may purchase VAPPs. For example, Govindasamy et al. [13] reported that consumers with high education and those without children tend to purchase VAPPs. In a more recent study, Melendrez-Ruiz et al. [14] reported that cultural and demographical differences underlie the formation of a distinct perception toward pulses’ consumption. Nevertheless, studies remain descriptive and have raised the need for an in-depth investigation of how consumers engage, form attitudes, and their behavioral intentions associated with VAPPs in the U.S. [10].

Considering that the purchase of VAPPs could be a planned consumption behavior, the theory of planned behavior (TPB) was seen as relevant to this study in providing a theoretical foundation. The TPB has been applied in various nutritional contexts such as organic vegetables [15] and wellbeing foods [16], but has not been utilized for functional foods such as VAPPs. A theoretical tool that successfully predicts intentions in a multitude of behavioral domains is assumed to be useful to understanding relevant attitudes and behavioral intentions of VAPPs.

Prior TPB models have emphasized cognitive components (i.e., attitude, subjective norm, and behavioral control) as determinants of purchase intention [12,17] yet have missed the role of emotions [18]. This may lessen the predictive power of the TPB in food consumption, which is not only a consequence of a rational or cognitive choice [19]. Integrating an affective component into the TPB model is an opportunity to enhance the model’s explanatory power in food preferences and consumption [20,21].

The positive effect of purchase intention on the consumer has been widely accepted in studies employing the TPB [22] and is somewhat intuitive. However, its effect on consumer behavioral intentions, such as word of mouth (WOM) and willingness to pay (WTP), in the agri-food context is uninvestigated. Furthermore, perceived barriers, such as price and market availability, may change the accepted relationships (intention → behavior), therefore need to be studied in influencing consumer attitudes and behavioral intentions towards VAPPs [23,24].

Given these, the purpose of this study is to extend a theoretical framework based on the theory of planned behavior (TPB) model [17] by including an affective factor (i.e., pride), perceived barriers, and subsequent consumer behavioral intentions (i.e., WOM and WTP) in the context of VAPPs. Particularly, WTP and WOM need to be studied for VAPPs because they are associated with a product provider’s ability to get its consumers [25]. This study seeks to answer the following questions: (1) Which construct of the TPB model explains the greatest variance in U.S. consumers’ purchase intentions of VAPPs? (2) Is pride an important factor in developing the purchase intention of VAPPs? (3) Can perceived barriers moderate the paths (purchase intention →WOM; purchase intention → WTP; subjective norm → purchase intention)? This study is meaningful to understand how U.S. consumers can engage in purchasing VAPPs—increased interest in VAPPs in the U.S. may not necessarily be in parallel to VAPPs’ purchase intention—and how purchasing barriers can influence or not influence their purchase intention for VAPPs.

## 2. Theoretical Background

### 2.1. Value-Added Product

Value-added products have been introduced to enhance diet quality by increasing availability and consumption [26] and involve the process of changing or transforming a product from its original state to a more valuable state [27,28]. Research focusing on VAPPs has been largely conducted in the context of developing countries such as India [1]. Recently, US pulse producers have looked for opportunities to increase the domestic consumption of pulses through value-added products [11]. However, to an extent, pulse studies have focused on investigating the functional properties of pulses and evaluating their nutritional benefits. Accordingly, the topic of the consumption behaviors of VAPPs still receives little attention overall [29], despite the increasing consumer demand for them [23].

In a recent study, Katoch [30] argued that the focus of value addition to agricultural products contributes to society by providing an opportunity for comprehensive development and improving the socioeconomic status of a rural community. It also creates job opportunities and brings income to farmers. This can be a reason that those value-added products are seen as the future profitability of agriculture [28]. Given this background, there is a greater need to understand how consumers purchase such products.

### 2.2. Theory of Planned Behavior

The premise of the TPB is that consumers are rational and motivated to make a decision, thereby making a reasoned choice among alternatives [17]. In general, research findings have indicated that the TPB consistently and accurately predicts individuals’ intentions and behaviors [31]. As such, the TPB is widely utilized in consumers’ decision-making processes in various contexts, including recently published studies on food consumption. For instance, Contini et al. [32] and Wang and Scrimgeour [33] employed the TPB to explain consumers’ purchase intentions and behaviors regarding plant-based foods. The central factor of the TPB is an individual’s intention to perform an actual behavior because intention captures one’s motivation to engage in behaviors [17]. Therefore, if one has a strong intention to perform a behavior, he/she most likely exert an effort to engage in that particular behavior. According to Ajzen [17], the intention is based on three conceptual determinants: attitude, subjective norm, and perceived behavioral control.

#### 2.2.1. Attitude

Attitude in the TPB refers to the degree to which an individual has a positive or negative evaluation of the behavior in question [34]. Specifically, attitude is a function of an individual’s behavioral beliefs regarding the perceived consequences of the behavior [35]; beliefs produce a positive or negative anticipated outcome or experience [36]. Therefore, the stronger the attitude toward a behavior, the greater the intention to perform the behavior.

Although an individual’s purchase intention is determined by the combination of attitude, subjective norm, and behavioral control, the role of attitude in developing consumers’ purchase intention toward foods is consistent [12]. For example, empirical evidence focusing on health benefits in food preferences indicates that attitude predicted the purchase intention. Sultan et al. [22] and Scalco et al. [37] found attitude to have the most significant contribution to purchase intention for organic foods. A similar result was found in research explaining the consumer decision-making process for soy products [38]. Consumer food selections are a routine behavior. However, consumers’ salient belief (i.e., attitude) in pulses is largely a health-promoting aspect [1,28]. This most likely enables consumers to anticipate favorable outcomes (i.e., enhancing health). In light of the above, this study posits the hypothesis that:

**Hypothesis** **1** **(H1).** *Attitude positively and significantly influences the purchase intention of VAPPs*.

#### 2.2.2. Subjective Norm

Subjective norm refers to the perceived social pressure regarding whether or not to perform the behavior and is associated with normative beliefs about whether others think he/she should engage in the behavior [17]. Normative beliefs include two types of beliefs: injunctive and descriptive normative beliefs [39]. Whereas the former belief is what people (e.g., friends and family) think or whether they approve of the behavior in question, the latter one is about how others themselves perform the behavior [34,37]. The TPB may highlight injunctive normative beliefs [37] because social influence in relation to food choice may come from important referent individual groups, such as family and friends [34]. In fact, Zagata [40] argued that a relevant source of social influence in consuming organic foods comes largely from family and friends. For example, the effect of subjective norm on purchase intention is strong in the context of plant-based diets [41] and fast food [42] among young people.

Furthermore, many studies have documented subjective norms as an important factor of intention in various food choice contexts, including organic food [37], fast food [42], and plant-based diets [33]. One’s subjective norm in food choice involves his/her desire to accommodate the opinions of salient referents for behavior [43], given that adhering to norms provides a chance to obtain social approval [44]. Studies have indicated that food preferences such as a plant-based diet (e.g., [33]) have a greater effect on the subject norm than attitude to the development of intention, and those food choices (plant-based diets and organic preferences) are more like a lifestyle greatly influenced by referent groups such as family and friends [45]. VAPPs consumption can also be considered as a food choice as per lifestyle [46]. Accordingly, we propose the following hypotheses:

**Hypothesis** **2** **(H2).** *Subjective norms positively and significantly influence the purchase intention of VAPPs*.

**Hypothesis** **2a** **(H2a).** *Of all the factors (i.e., attitude, subjective norm and behavioral control) influencing the purchasing intention of VAPPs, subjective norms have the most significant impact*.

#### 2.2.3. Perceived Behavioral Control

Perceived behavioral control depends on an individual’s perception of how easy or difficult it is to engage in the behavior; thus, it is conceptually similar to self-efficacy [17,47]. However, while self-efficacy focuses on internal factors (e.g., confidence or skills) to execute a behavior [48], behavioral control reflects the presence of external factors as well (e.g., time) that can improve or impede behavior performance [49]. According to Ajzen [17], internal and external factors are conceptually independent. For instance, one may purchase VAPPs knowing their environmental benefits (internal factor), or because they are readily available (external factor). When individuals believe that they have resources and opportunities, with few obstacles, they are confident in their ability to perform the behavior; therefore, they exhibit a high degree of perceived behavioral control [12].

A number of studies have contended that one’s ability to control and perform a behavior positively influenced the individual’s intention. For example, Han et al. [50] found that perceived behavioral control influenced people’s intention to visit a green hotel. Furthermore, Aertsens et al. [51] found a direct impact of perceived behavioral control on behavior when the behavior is perceived as difficult. There are some impediments to purchasing VAPPs, such as accessibility or high price [23,24,46]. These difficulties to get the products may motivate the purchase intention of the consumers.

Other studies have supported the similar effect of perceived behavioral control on intention in various contexts such as recycling [52], using a green hotel [53], and food choice [54]. Such behaviors are indifferent to VAPP consumption in terms of volitional behavior that may require internal and external factors to perform the behavior. The following hypothesis was thus postulated:

**Hypothesis** **3** **(H3).** *Behavioral control positively and significantly influences the purchase intention of VAPPs*.

### 2.3. Pride

Although the TPB has been applied to a range of studies in food products, the main interest remains the function of cognitive factors (attitude, subjective norm, and behavioral control) leading to intention. Researchers have argued that emotions are linked to a consumer’s decision-making process and increase explanatory power, as seen in certain behavioral models [20,21,55]. The role of emotion has been overlooked in TPB models, however, since it is typically a cognitive-based model [21]. Focusing on cognitive components only in explaining the decision-making process fails to understand consumer behavior [56].

This study focuses on pride primarily in regard to the purchasing intention of VAPPs because pride is an emotion related to the performance of volitional behavior and self-control [57]; therefore, it is cognition-dependent [58]. Accordingly, pride can conceptually accompany models emphasizing cognitive elements, such as the TPB, in explaining behaviors. The findings on the roles of pride in product consumption are limited while suggesting that experiencing pride should positively influence consumers’ purchase intention [59].

Pride is presented as a feeling of satisfaction or pleasure derived from goal achievement [60]; therefore, it is a self-conscious emotion [61]. Studies have argued that pulses consumers in the U.S. are a health-conscious group [28], and anecdotal evidence suggests that the group of consumers may get motivated to purchase VAPPs by experiencing positive emotions because VAPPs signal to satisfy their concerns about health.

Additionally, pride has been extensively used in the selection of sustainable products [62], and enables consumers to make conscious decisions [63]. In the literature on consumer behavior, consumption meeting personal standards or values has been found to engender positive emotions such as pride [64]. The purchase of VAPPs can be framed as a conscientious behavior rather than one of emotional resonance. Given these notions, it seems reasonable to speculate that pride motivates VAPP consumption, as a health-focused diet requires self-control and a conscientious selection of foods. Thus, we propose the hypothesis that:

**Hypothesis** **4** **(H4).** *Pride positively and significantly influences the purchase intention of VAPPs*.

### 2.4. Word of Mouth and Willingness to Pay

Word of mouth (WOM) refers to informal communication between consumers concerning evaluations of goods and services [65], rather than formal messages to firms or service personnel [66]. WOM includes positive, negative, or neutral formats [66]. Many studies contend that WOM is a powerful force in the marketplace [67] because informal information strongly influences consumer evaluation of products and services [68]. A pleasant experience, for instance, can result in the consumer recommending the product to others, while product defects can lead to the sharing of complaints and frustration with others. WOM provides vital information to consumers and helps them decide whether a product or company deserves loyalty [69]. In this sense, a company can benefit from positive WOM.

Studies consistently support a positive relation between purchase intention and WOM. Yasin and Shamim [70] found that the purchase intention of a brand positively influenced WOM. In the VAPPs context, we particularly propose such a relationship (purchase intention → WOM) because consumers may want to prove their conformation to a referent group by talking about the product, implying that the subjective norm indirectly influences WOM regarding VAPPs. In the theoretical argument of the TPB, a consumer’s positive WOM is more likely to increase once their purchase intention increases. Therefore, the positive effect of the intention to purchase VAPPs on WOM is suspected.

Willingness to pay (WTP) corresponds to the maximum amount a customer is willing to pay for a product or service [71] and has been largely investigated for green or pro-environment products and behaviors. For a value-added product, consumers were willing to pay 30% more [72]. In the context of food preferences, factors leading to WTP include food-related lifestyles and food quality. For instance, health-conscious consumers showed high WTP for green restaurants [73,74], and nutrition positively affects WTP when purchasing value-added products [75].

In the theoretical domain of the TPB, the components (e.g., health consciousness) can be translated as an underlying condition that contributes to forming attitudes and subjective norms. Nutrition and freshness explain the expected consequences for consumers. This creates a positive attitude towards the product, leading to a positive purchase intention and WTP. Similarly, lifestyle may function to form injunctive normative beliefs, associated with the subjective norm connecting to intention and WTP. The following hypotheses were hence proposed:

**Hypothesis** **5** **(H5).** *Purchase intention positively and significantly influences WOM for VAPPs*.

**Hypothesis** **6** **(H6).** *Purchase intention positively and significantly influences WTP for VAPPs*.

### 2.5. Perceived Barriers

Perceived barriers can be defined as a person’s perceived obstacles to performing a particular behavior [76]. Researchers in marketing and consumer behavior have indicated the impact of perceived barriers on consumers [77,78]. Similarly, this study focuses on perceived barriers as a moderator because perceived barriers are an important situational factor that can change product evaluation [79]. Perceived barriers explain why consumers wanting to engage in consumption ultimately decide not to purchase a product [77]. Therefore, they can weaken or reverse the positive effect of intention on consumers’ behavior.

Many service marketing researchers agree that perceived barriers moderate the formation of consumers’ intentions and behaviors [76]. For instance, Jones et al. [80] found that perceived barriers moderated the relationship between satisfaction and behavioral intentions, in that the level of perceived barriers either weakens or strengthens the relationship. Similarly, empirical evidence shows that perceived barriers have a moderating effect on the relationship between intention and its antecedents [78,81].

Consistent with the definition of perceived barriers, the present study interprets perceived barriers in the context of VAPPs as hindering factors that may make it difficult for consumers to purchase VAPPs. Perceived price and insufficient product availability in the markets are perceived barriers to VAPPs [23,24,46], and most likely inhibit consumers from purchasing VAPPs. The perceived high price of VAPPs could be a possible reason why consumers who have disposable incomes prefer to purchase VAPPs [13]. Insufficient product availability may lead consumers to other products that can be easily accessed, thereby weakening the effect of purchase intention on behaviors. The relationship between intention and behavior is contingent on the level of perceived barriers in the VAPP market.

**Figure 1:**
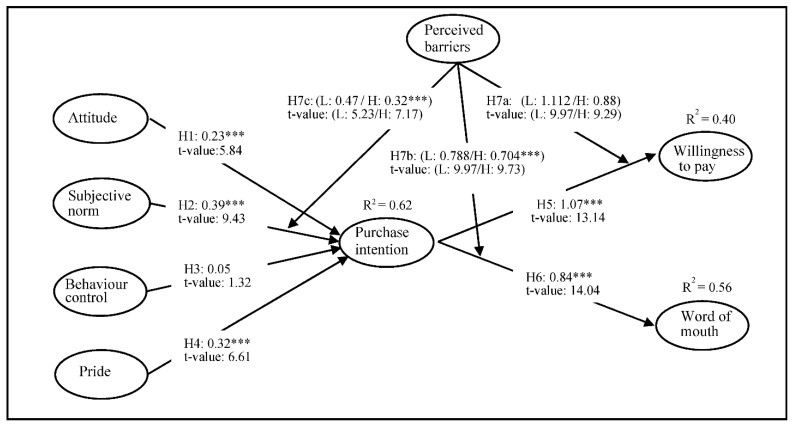
Results of the Structural Equation Modeling. Note: *** *p* < 0.001.

Among studies using the TPB, research investigating the factors that may weaken the positive influence of attitude, subjective norms, and behavioral control on intention is lacking. As discussed in the previous section, the subjective norm is possibly the most significant component that leads to purchase intention in VAPP consumption. However, if the consumer has high barriers to purchasing VAPPs, her/his confidence in carrying out the intention to purchase VAPPs most likely gets discouraged, regardless of how referent groups expect her/him to perform a certain behavior. In this case, perceived barriers may overshadow the relationship between subjective norms and intentions. Therefore, perceived barriers inhibit a particular intention to perform, and consumer behavior is associated with intention [82]. Describing shopping behavior, Han et al. [83] found that consumers’ perceived disadvantages threatened the relationship between behavioral intention and its predictor. Therefore, perceived barriers may moderate the relationship between subjective norms and purchase intentions. We, therefore, propose the following hypotheses (see Figure 1):

**Hypothesis** **7a** **(H7a).** *Perceived barriers moderate the positive influence of intention to WTP such that the positive influence of purchase intention on WTP is weaker for consumers who have a high level of perceived barriers*.

**Hypothesis** **7b** **(H7b).** *Perceived barriers moderate the positive influence of intention to WOMsuch that the positive influence of purchase intention on WOM is weaker for consumers who have a high level of perceived barriers*.

**Hypothesis** **7c** **(H7c).** *Perceived barriers moderate the positive influence of subjective norm on purchase intention such that its positive impact is weaker for consumers who have a high level of perceived barriers*.

## 3. Methods

### 3.1. Survey Development

The survey questionnaire consisted of three sections: study description, inquiries for the evaluation of research constructs, and requests for demographic information. The measurement items were selected from well-established research across multiple disciplines [84,85,86,87,88]. The content of the adopted items was modified to fit the present research setting. Specifically, four items were used to measure attitude and behavioral control, and three items for subjective norm, pride, purchase intention, WTP, and WOM, respectively. The variable of perceived barriers was assessed using two items. The measures used were a 7-point Likert scale.

The adopted items were reviewed and revised by two researchers to avoid content ambiguity. The questionnaire was checked by industry experts for minor modification and reality and then imported into Qualtrics. Twelve students reviewed and checked the developed questionnaire in Qualtrics for readability and technical flow. Based on their feedback, the questionnaire was further improved and finalized. The developed surveys were distributed to consumer panels in the U.S. recruited by a market research company, Dynata, based in Connecticut. The company sent the survey to its consumer panels twice; for the second time, consumers who received the survey already were excluded to ensure only one participation. The data consisted of 321 respondents from the first data collection and 466 from the second data collection. To check non-response bias, the mean responses of those two groups on selective survey items were compared. There were no statistically significant differences. Therefore, non-response bias was not a concern of the sample. The measures used in this study are presented in Table 1.

### 3.2. Data Collection and Analysis

The designed questionnaires were distributed to randomly selected U.S. consumers through an online marketing research company. A total of 787 questionnaires were received, and 296 usable responses remained after refining the dataset, indicating a 37.61% valid response rate. Of the respondents, 47.3% (*n* = 140) were male and 52.4% (*n* = 155) were female. Their ages ranged from 18 to 73, showing an equal response rate (*n* = 89, 30.1%) of consumers in Generation X and Baby Boomers. Amos 25 with the Maximum likelihood estimation function was used. This study employed a two-step approach [89]: a measurement model was first estimated using Confirmatory Factor Analysis (CFA), and a structural model was established to test the proposed causal relationships. To test the moderation effect, measurement invariance was conducted; for this, mainly χ^2^ was compared between a fully constrained model and a constrained model.

## 4. Results

### 4.1. Data Screening

The normality assumption was checked to perform the SEM. The results showed that (a) the skewness values of the latent variables were near zero, and (b) the kurtosis values remained within the acceptable range between −2 and +2 [90]. The KMO values for sampling adequacy were between 0.70 and 0.94, and the Bartlett test of sphericity index for linearity was statistically significant (*p* < 0.001) for each construct. Therefore, it was concluded that the normality and factorability assumptions for SEM were met. The reliability of each construct ranged from 0.91 to 0.96.

### 4.2. Measurement Model

Confirmatory factor analysis was conducted with all indicators using the maximum likelihood estimation. To increase reliability and decrease measurement error [91], those with weak loadings (lower than 0.6) were deleted, and items with standardized residual covariance value of 0.4 or above were eliminated [92]. During this process, two items were deleted (AT1 and BC4). The remaining 23 items were subjected to the CFA, the results of which showed an acceptable fit [93] to the data (*cmin/df* = 2.385; CFI = 0.962; RMSEA = 0.069; GFI = 0.872; NFI = 0.937; TLI = 0.952; AGFI = 0.825). It was hence concluded that the measurement model is suitable for predicting the overall data.

#### Measurement Validity

Convergent and discriminant validity were assessed to examine the extent to which measures of latent variables shared their variance and how they differed from others [94]. Convergent validity is established when the average variance extracted (AVE) of each construct is greater than 0.50 [95], while discriminant validity is confirmed when the maximum shared variance (MSV) is smaller than the AVE [95]. The AVE of each construct was measured and compared to the inter-factor correlations. As shown in Table 2, convergent and discriminant validity were confirmed. All AVE values ranged from 0.588 to 0.873. Construct reliability was also checked by estimating composite reliability. The composite reliability scores ranged from 0.737 to 0.954. To calculate CR and AVE, Excel was used, and composite reliability was reported because Cronbach’s alpha is a lower bound to reliability thereby underestimating true reliability [96].

### 4.3. Structural Model

The model fit was examined against the thresholds suggested by Hu and Benter [93]. It showed an adequate fit to the data (*cmin/df* = 2.698, CFI = 0.959, RMSEA = 0.076, GFI = 0.872, NFI = 0.936, TLI = 0.950, AGFI = 0.817). The R^2^ scores for endogenous variables (R^2^_purchase intention_ = 0.62, R^2^_WTP_ = 0.40, and R^2^_WOM_ = 0.58) were moderate.

#### Hypotheses Testing

The standardized coefficients showed that the proposed relationships were statistically significant except for the path from behavioral control to purchase intention: attitude → purchase intention (β = 0.23, *p* < 0.001); subjective norm → purchase intention (β = 0.386, *p* < 0.001); and pride → purchase intention (β = 0.357, *p* < 0.001), supporting H1, H2, and H4. The results also revealed that purchase intention had a positive influence on WTP (β = 1.082, *p* < 0.001) and WOM (β = 0.859, *p* < 0.001). Thus, hypotheses 5 and 6 were supported. This indicates that (a) attitude, subjective norm, and pride are positively associated with the purchase intention of VAPPs, and (b) purchase intention of VAPPs leads to WOM and WTP, such that when consumers show high purchase intention, they are more likely to pay more for VAPPs and to spread WOM regarding the products (e.g., recommending them to friends). However, the proposed impact of behavioral control on purchase intention was insignificant (β = 0.053, *p* > 0.05), indicating that H3 was not supported. Figure 1 shows the results of the proposed model with coefficients and t-values, and Table 3 presents the structural parameter estimates of each path.

### 4.4. Measurement Invariance

Prior to assessing the measurement model, a common method bias (CMB) was checked. CMB was examined, using Harman’s single factor score, which is considered the simplest way to test CMB [97]. The unrotated principal axis factoring analysis showed a single factor, explaining 46.2% of the total variance. It was concluded that CMB was not a critical concern in this study.

A metric invariance test was conducted to evaluate the moderating impact of perceived barriers on the relationship between purchase intention, WOM, and WTP. A median split (*Mdn* = 4) approach was used to divide the responses into two groups: a low level of perceived barrier group (152 cases) and a high level of perceived barrier group (144 cases). A baseline model comprising these two groups was developed. A visual inspection of the unconstrained model suggested a configural invariance by showing an adequate fit to the data (χ^2^ (404) = 802.3, *p* < 0.001, χ^2^/df = 1.98, CFI = 0.943, RMSEA = 0.058, GFI = 0.807, NFI = 0.892, TLI = 0.928; AGFI = 0.736). To confirm the invariance, a metric test was conducted. For this purpose, the baseline model is fully constrained. The constrained model generated an adequate fit to the data (χ^2^ (427) = 826.6, *p* < 0.001, χ^2^/df = 1.93, CFI = 0.941, RMSEA = 0.057, GFI = 0.805, NFI = 0.888, TLI = 0.930; AGFI = 0.732). Comparing these two models using the chi-square test, this study met the metric invariance test; the two groups were invariant regardless of their factor structure (χ^2^_diff_ = 24.3, *p* = 0.387).

### 4.5. Multigroup Analysis

To test H7_a_, H7_b,_ and H7_c_ (i.e., the moderating effect of perceived barriers), multigroup analysis was employed using the multigroup analysis feature of AMOS. The chi-square difference confirmed that the two groups were invariant. The results showed that the path from purchase intention to WOM was moderated by perceived barriers (β_low_: 0.890 → β_high_: 0.704, *p* < 10), thereby supporting H7_b_. That is, the influence of purchase intention on WOM was weakened for consumers who had high perceived barriers.

The results also showed that the path from the subjective norm to purchase intention (H7c) was significantly different between the high and low perceived barrier groups (β_low_: 0.324 → β_high_: 0.481, *p* < 10), thereby supporting H7c. The data analysis rejected H7_a_; perceived barriers did not moderate the impact of purchase intention on WTP (β_low_: 1.113 → β_high_: 0.908, *p* > 10). The results of the multigroup analyses are presented in Table 4. The hypotheses proposed by this study and the results are summarized in Table 5.

## 5. Discussion

This study investigated the empirical evidence for the relationships among the TPB variables (i.e., attitude, subjective norm, and behavioral control) and pride, and tested the role of perceived barriers within an expanded TPB model. Little is known about the decision-making process for purchasing VAPPs. To the best of our knowledge, this study is one of the first attempts to use the TPB in the context of pulse consumption. Further, it is one of the very few studies that expand the TPB by incorporating an emotional factor.

### 5.1. Sufficient Relationships

In line with existing studies on food selection, the subjective norm has the strongest impact on the purchase intention of VAPPs. For example, Scalco et al. [37] documented the strongest impact of subjective norms on intention to use organic foods. Consistent findings have been reported in the domain of fast food [42] and plant-based food [33] as well. We predicted that subjective norm was the strongest predictor of VAPP purchase intention because food choice frequently is greatly influenced by others [37]. Furthermore, when consumers do not have enough experience or knowledge of food products, they are open to others’ opinions [98]. Family and friends are easily a source of encouragement or discouragement in selecting food items and making a dietary change. This may also explain why WOM was strongly predicted by intention derived from subjective norms. By nature, VAPPs are possibly more attractive to health-conscious consumers—a relatively specific consumer group—than those who are not. Throughout the interactions with a similar mind, giving information (i.e., WOM) on VAPPs may foster a sense of belonging; WOM could be increased when there are health and other benefits related to the foods.

Even though it was not the strongest predictor as other food studies presented (e.g., [22,37,38]), attitude explains the purchase intention of VAPPs. The attitude construct in the TPB is closely related to the expectation of gaining benefits; therefore, the positive effect of attitude was somewhat intuitive in VAPPs because the nutritional benefits of pulses are well known [9]. The result of this study can be explained from the perspective of product familiarity and availability because they are both associated with consumers’ attitudes toward foods [99]. When consumers have sufficient knowledge and experience with a food product, attitudes (either positive or negative) can be developed. However, in the case of VAPPs, consumers have not had the opportunity to become familiar with them [100]. Therefore, it could be speculated that attitude could be the strongest predictor of purchase intention if VAPP market availability meets consumer demand.

This study suggested the critical role of pride in the purchase intention of VAPPs; pride was the second most influential factor in the purchase intention of VAPPs. Although it was contrary to the assumption of sufficiency [34], adding pride to the existing TPB variables was appropriate in relying on the assertion that a conceptually independent construct can be added to existing predictors of the TPB [34]; CFA confirmed that pride and other constructs were distinctive. While pride is a self-conscious emotion [61] that plays an important role in food consumption, it has rarely been used to explain food consumption. This study reveals that pride is an influential predictor of the intention to purchase VAPPs. The role of pride in the selection of food products, particularly healthy and environmentally friendly food products, is significant because consumption that may meet personal standards or values elicits positive emotions [64]. Our results are somewhat consistent with those of Kim and Huang [62], who argue that pride is involved in the decision-making process for local food consumption. Although its role in their study was different (pride was a dependent variable within the study), it is important to understand that pride plays a critical role in food consumption.

We explain its impact on purchase intention from the perspective of feelings of achievement and self-awareness [101]. Purchasing VAPPs may involve self-awareness behaviors because products such as VAPPs are intentional, and even somewhat inconvenient, to buy. When self-awareness plays a role, pride motivates people to pursue their goals [101], which strengthens purchase intention and behavior. Further, pride presents satisfaction or pleasure; such self-motivated satisfaction strongly motivates the intention to perform a behavior under consideration [60].

#### Supported Moderating Effect

Even though the effect of perceived barriers on WOM was somewhat intuitive, we find a possible explanation for the impact of perceived barriers on relational changes (subjective norm → purchase intention → WOM) from equity theory. Based on equity theory, consumers may feel greater sacrifices or investments to purchase VAPPs as there are perceived barriers, which mean extra work to handle or buy them [87]. In this sense, the cost of obtaining VAPPs may overpower the positive associations among the variables, weakening the relationships.

Perceived barriers moderate the effect of subjective norms on purchase intention. This result explains social influences on the choice of food products. The concept of subjective norm embraces “relational factors”, for instance, social pressure [17]. When, for example, friends and family perceive some of the barriers to consuming VAPPs, their opinions abate one’s purchase intention for VAPPs because people may want to comply, either consciously or subconsciously, with social norms (i.e., others’ opinions). Information coming from a referent group may also mean more reliable and credible—for instance, my family wants me to be well—influencing the purchase intention of VAPPs.

### 5.2. Insufficient Relationships

Unlike existing TPB studies, the impact of behavioral control on the purchase intention of VAPPs was found to be insignificant in this study. However, according to Ajzen [12,17], the roles of the three variables in the TPB may vary across situations. Furthermore, because behavioral control denotes the subjective degree of control over behavior performance [34], potential VAPP consumers may not feel a degree of control due to the present lack of access to VAPPs [23,24,46]. Our finding is consistent with other food selection studies (e.g., [18,102]). For example, Mahon et al. [102] argued that when consumers with a high level of confidence, behavioral control may not influence their purchase intention. Kim et al. [18] suspected that individuals would find it difficult to predict behavioral control relating to their future behaviors. These findings could be possible because VAPPs purchase may not require strong perceived control to perform; they could be a simple transaction-like performance, meaning that consumers may not act on their intention when a situation does not present the chance to perceive behavioral control [103]. It could also be argued that food consumption is a habitual behavior that may not require the ability to perform a behavior [104].

However, consumers are unlikely to recommend VAPPs when they feel barriers. Interestingly, perceived barriers did not moderate the path from purchase intention to the WTP. This could mean that value-added pulse products may equal an expensive product to consumers; general perceptions toward VAPPs may be “already pricy,” [23,24,46], making perceived barriers, such as high price, ineffective.

## 6. Conclusions

Research regarding value-added pulse products has been limited overall in food selection and consumption. To overcome this shortcoming, the present study investigates US consumers’ decision-making processes associated with VAPPs by incorporating pride and perceived barriers into the TPB. Furthermore, given the sparse amount of empirical work on value-added products, this study has various implications for researchers and agri-food producers, and marketers focusing on the US market. The biggest question that agri-food producers need to answer is what do customers really value. It is important to understand this before a value-added product reaches a competitive market. This study provides insights into what US consumers value from using VAPPs.

The following critical points were presented in the study: (a) subjective norm has the strongest effect on the purchase intention of VAPPs; (b) pride significantly determines the purchase intention of VAPPs; (c) perceived barriers moderate positive relationships between subjective norm and purchase intention, and between purchase intention and WOM; and (d) behavioral control does not significantly influence purchase intention, unlike other studies that show a positive and strong relationship between the two variables. Rather, our findings show that decision-making models become sophisticated, and consumers’ purchase intentions and behaviors are complex. Many underlying psychological factors need to be studied to benefit the agri-food business.

## 7. Theoretical Implications

To the best of our knowledge, this is the first empirical study to investigate the decision-making process for value-added pulse products. Studies related to pulses in the context of agri-foods have been largely lacking in understanding consumer behavior. By extending the TPB, this study contributes to the development of theories in the context of agri-foods and understanding of consumers’ behaviors toward pulses and value-added pulses products. This research is one of the very few studies that combines an emotional component to the TPB model. Particularly, pride predicted the purchase intention of VAPP stronger than attitude and behavioral control. This suggests that the consumption of health- or functional-food products is not all about a rational or cognitive choice [19] yet rather reflects the complex mechanisms of purchase intention with functional-food products. Despite the research context being value-added pulse products, the tested model can be used for other contexts as well, such as diet. Furthermore, this study considers behavioral intentions (i.e., WOM and WTP) in agri-food products; this could increase the reality aspect of this study for the case of not measuring actual consumption behaviors. This study provides evidence to help producers and marketers of agri-foods attract consumers, thereby adding a new perspective to agri-food literature. Additionally, it provides theoretical explanations of how to respond to consumers. Some existing research has shown that attitude is a critical factor in food selection. However, this may not be applicable to VAPP consumption.

## 8. Industry Implications

This study suggests that producers and marketers of pulses use positive emotions such as pride to promote pulse consumption. A marketing campaign evoking pride in pulse consumption will be attractive to consumers. For example, “pride in pulse” or “pride in local value-added pulse products” may generate attention from consumers, given the considerable recent attempts to promote local food. Agri-food businesses should find a way to influence a referent group(s) for marketing VAPPs. Recently, young generations are getting interested in healthy food products. Marketing strategy using peers’ recommendations may be an effective way to consider. Educational marketing campaigns, such as the benefits from VAPPs, could be a considerable plan to increase VAPPs; presenting the specific health and/or environmental aspects of VAPPs through multiple information sources could improve positive attitudes toward VAPPs. Offering information sessions targeting students or young generations may foster prospective consumers. There is evidence indicating that young generations will pay close attention to healthy and functional foods after the COVID-19 pandemic. Therefore, the “better-for-you” claim may appeal to those young generations. In addition, the agri-food industry should consider developing a meal kit(s) that has been trendy in foods; this type of food product may increase pulses’ consumption as well as VAPPs in making consumers feel it is easy to handle and to cook healthy food.

It is suggested that industries and state governments need to make efforts to eliminate perceived barriers to encourage VAPP consumption, including general pulse consumption. Plans to eliminate perceived barriers should be organized and implemented by a collaboration of producers, marketers, and policymakers because such concerns require multiple aspects. For example, marketers should make more efforts to disseminate benefits and products related to pulses. Disseminating information regarding VAPPs can be conducted in schools by actively communicating with them.

To make VAPPs more visible in markets, producers and policymakers can consider bringing various stakeholders together in various forums, for the purposes of sharing information and working together [5]. The efforts of stakeholders to minimize the perceived barriers (e.g., high price and product availability) will increase VAPPs’ consumption, thereby increasing pulse consumption.

## 9. Future Research and Limitations

Recent work in food selection has greatly increased the understanding of the TPB, but, until now, the decision-making process for food selection has yet to adopt emotions such as pride. Researchers may consider including various emotions to explain the attitude and behavioral intentions in the context of food selection and consumption. Furthermore, other situational factors such as perceived benefits and knowledge can be used to test whether they strengthen or weaken the positive relationships among variables in a decision-making model for food selection. An investigation of the moderating effect of pride on each path will be interesting to argue the importance of emotion for purchase intention in the field of healthy and functional food. The path from behavioral control to purchase intention was not supported. We suspected that using self-efficacy instead of behavioral control may present a different outcome; therefore, researchers may consider using self-efficacy in explaining the consumption of pulses or VAPPs.

This study has some limitations, such as the limited sample. Given that our sample was only from the United States, and that various factors influence human behavior [105], the findings of this study should not be generalized. Furthermore, although the findings reflect theoretical support, external validity may be a concern, because we used a self-reported survey. However, this concern is not unusual, pertaining to any study using surveys. The coefficient value from purchase intention to a willingness to pay was greater than one, implying multicollinearity. However, the two variables are conceptually very similar, and the primary intention for the particular path was to make a prediction, not to understand the effect of purchase intention on willingness to pay.

## Figures and Tables

**Table 1:** Measurement model results.

Measures	Factor Loading	CR	AVE
Attitude		0.951	0.866
To purchase a value-added pulse product (e.g., lentil paste, black bean chips) is			
• Undesirable/Desirable	0.89		
• Unfavorable/Favorable	0.95		
• Unlikeable/Likable	0.95		
Subjective norm		0.954	0.873
• Most people who are important to me think I should choose a value-added pulse product (e.g., lentil paste, black bean chips) when grocery shopping.	0.92		
• Most people who are important to me would want me to choose a value-added pulse product (e.g., lentil paste, black bean chips) when grocery shopping.	0.94		
• People whose opinions I value would prefer me to choose a value-added pulse product (e.g., lentil paste, black bean chips) when grocery shopping	0.94		
Behavioral control		0.935	0.827
• I am confident that if I want, I can choose a value-added pulse product (e.g., lentil paste, black bean chips) when grocery shopping.	0.92		
• I am capable of choosing a value-added pulse product (e.g., lentil paste, black bean chips) when grocery shopping.	0.94		
• I have enough resources (money) to choose a value-added pulse product (e.g., lentil paste, black bean chips) when grocery shopping.	0.97		
Pride		0.939	0.837
• How intensely would you feel pleased?	0.92		
• How intensely would you feel good about yourself?	0.93		
• How intensely would you feel pride?	0.90		
Purchase intention		0.942	0.845
• PI 1: I am planning to purchase a value-added pulse product (e.g., lentil paste, black bean chips) in the future.	0.94		
• I intend to purchase a value-added pulse product (e.g., lentil paste, black bean chips) when grocery shopping in the future	0.94		
• I will make an effort on finding a value-added pulse product (e.g., lentil paste, black bean chips) when grocery shopping in the future	0.89		
Word-of-Mouth (WOM)		0.918	0.788
• I’m likely to say good things about a value-added pulse product (e.g., lentil paste, black bean chips).	0.88		
• I would recommend a value-added pulse product (e.g., lentil paste, black bean chips) to my friends and relatives.	0.92		
• I recommend a value-added pulse product (e.g., lentil paste, black bean chips) to others	0.82		
Willingness to pay (WTP)		0.910	0.771
• I would pay more to purchase a value-added pulse product (e.g., lentil paste, black bean chips) at a store.	0.91		
• I would be willing to pay an extra percentage of my receipt if I choose a value-added pulse product (e.g., lentil paste, black bean chips) when shopping.	0.90		
• There is a strong likelihood that I would pay more if I choose a value-added pulse product (e.g., lentil paste, black bean chips) when grocery shopping.	0.82		
Perceived barriers		0.737	0.588
• To purchase a value-added product (e.g., lentil paste, black bean chips) means to pay more.	0.86		
• To purchase a value-added product (e.g., lentil paste, black bean chips) means to have difficulties in finding them on the market.	0.66		

Note: CR-composite reliability, AVE-average variance extracted.

**Table 2:** Convergent and discriminant validity.

	MSV	MaxR(H)	WOM	AT	SN	BC	PR	PI	WTP	PB
WOM	0.656	0.921	0.888							
AT	0.462	0.957	0.680	0.931						
SN	0.667	0.955	0.722	0.461	0.934					
BC	0.425	0.941	0.566	0.652	0.438	0.909				
PR	0.656	0.940	0.810	0.638	0.695	0.592	0.915			
PI	0.653	0.947	0.808	0.676	0.634	0.619	0.718	0.919		
WTP	0.667	0.918	0.777	0.610	0.817	0.531	0.760	0.730	0.878	
PB	0.287	0.780	0.535	0.290	0.536	0.193	0.506	0.369	0.493	0.767

Note: MSV—maximum shared variances; MaxR—maximum reliability; Diagonal values denote square root of AVE and off-diagonal values represent correlation coefficients between constructs. WOM—Word of mouth; AT—Attitude; SN—Subjective norm; BC—Behavioral control; WTP—Willingness to pay; PB—Perceived barriers; PR—Pride; PI—Purchase intention.

**Table 3:** Structural parameter estimates.

Path	Std Estimate	S. E	t-Value
Attitude → Purchase intention	0.229	0.040	5.768 ***
Subjective norm → Purchase intention	0.386	0.041	9.432 ***
Behavioral control → Purchase intention	0.053	0.040	1.323
Pride → Purchase intention	0.357	0.054	6.617 ***
Purchase intention → Willingness to pay	1.082	0.071	15.169 ***
Purchase intention → Word-of-mouth	0.859	0.054	16.046 ***

Note: *** *p* < 0.001.

**Table 4:** Multigroup analysis results.

		Low Barriers			High Barriers			Z-Score
		Coefficient	t-Value	S.E	Coefficient	t-Value	S.E	
H7a	Purchase intention → Willingness to pay	1.113	9.874	0.113	0.908	10.298	0.088	1.429
H7b	Purchase intention → Word of mouth	0.890	9.988	0.089	0.704	10.730	0.066	−1.678 *
H7c	Subjective norm → Purchase intention	0.481	5.236	0.062	0.324	7.717	0.062	−1.796 *

Note: * *p* < 0.10.

**Table 5:** Hypotheses and results.

	Hypothesis	Result
H1	Attitude positively and significantly influences the purchase intention of VAPPs	support
H2	Subjective norms positively and significantly influence the purchase intention of VAPPs	support
H2a	Of all the factors (i.e., attitude, subjective norm, and behavioral control) subjective norms have the most significant impact	support
H3	Behavioral control positively and significantly influences the purchase intention of VAPPs	not support
H4	Pride positively and significantly influences the purchase intention of VAPPs	support
H5	Purchase intention positively and significantly influences WOM for VAPPs	support
H6	Purchase intention positively and significantly influences WTP for VAPPs	support
H7a	Perceived barriers moderate the positive influence of intention to WTP such that the positive influence of purchase intention on WTP is weaker for consumers who have a high level of perceived barriers.	not support
H7b	Perceived barriers moderate the positive influence of intention to WOM such that the positive influence of purchase intention on WOM is weaker for consumers who have a high level of perceived barriers.	support
H7c	Perceived barriers moderate the positive influence of subjective norm on purchase intention such that its positive impact is weaker for consumers who have a high level of perceived barriers.	support

Note: WOM denotes word of mouth and WTP denotes willingness to pay.

## Data Availability

This study did not report any data.
